# Phenotypic and genotypic characterization of biofilm-forming *Escherichia coli* from subclinical bovine mastitis and association with antimicrobial resistance

**DOI:** 10.1186/s12917-026-05598-2

**Published:** 2026-06-03

**Authors:** Hanan S. Khalefa, Asmaa M. Ali, Heba S. Farag, Mohamed S. Kamel, Amani Abd El–latif Mosleh, Noha M Bakry

**Affiliations:** 1 Department of Veterinary Hygiene and Management Faculty of Veterinary Medicine, Cairo University, Giza, 12211 Egypt; 2 Department of Medicine and Infectious Diseases, Faculty of Veterinary Medicine, Cairo University, Giza, 12211 Egypt; 3 Animal Health Research Institute, Shebin El-Kom branch, ARC, Shibin El-Kom, Menoufia 32511 Egypt

**Keywords:** Antimicrobial resistance, Biofilm formation, *E. coli*, Multidrug resistance, Phylogroup, Subclinical mastitis, Virulence genes

## Abstract

**Background:**

Bovine mastitis is one of the most significant and costly diseases in the dairy industry, and *Escherichia coli* (*E. coli*) is a common etiological agent of subclinical mastitis. This study aimed to phenotypically and genotypically characterize biofilm-forming *E. coli* isolated from subclinical bovine mastitis and to investigate their association with antimicrobial resistance. A total of 254 composite milk samples were collected from cows with subclinical mastitis in five dairy farms in Egypt.

**Results:**

The overall prevalence of *E. coli* was 26.7% (68/254), with the highest isolation rates in Giza (37.1%) and Cairo-Alexandria (30.2%) regions. Molecular screening of diarrheagenic *E. coli* (DEC) virulence genes revealed the presence of *st* (10.3%), *stx*_*2*_ (5.9%), and *eae* (4.4%), whereas all isolates were negative for *stx*_*1*_. All isolates harbored the biofilm-associated *luxS* gene, and 80.9% of isolates also carried *fimH*. Based on virulence gene profiles, 7.4%, 4.4%, 2.9%, and 2.9% of isolates were classified as ETEC, EPEC, STEC, and hybrid ETEC–STEC (ETST), respectively, while 82.4% were DEC-negative. Phylogrouping showed a predominance of group B1 (51.9%), followed by group A (35.3%). Antimicrobial susceptibility testing revealed high resistance rates to amoxicillin/clavulanic acid (60.3%) and cefuroxime (58.8%), whereas all isolates were susceptible to cefepime, and most were susceptible to enrofloxacin (95.6%) and nalidixic acid (94.1%). Multidrug resistance was detected in 52.9% of isolates, mainly involving resistance to amoxicillin/clavulanic acid, ampicillin, tetracycline, and sulpha/trimethoprim. Biofilm formation assessed using the microtiter plate assay showed that 44.1% of isolates were moderate and 38.2% were strong biofilm producers, with significant variation between farms (*p* < 0.0001). Hierarchical clustering and chi-square analyses indicated that biofilm strength was associated with resistance to selected beta-lactams (amoxicillin/clavulanic acid, ampicillin, and cefuroxime), phylogenetic background (especially groups A and B1), and farm origin, whereas MDR status and most virulence genes showed weak or no association.

**Conclusion:**

These findings highlight the important contribution of environmental and commensal *E. coli* lineages to subclinical mastitis in Egypt and underscore the need for improved farm hygiene and prudent antimicrobial use to limit the spread of biofilm-forming and antimicrobial-resistant *E. coli* in dairy herds.

**Supplementary Information:**

The online version contains supplementary material available at 10.1186/s12917-026-05598-2.

## Background 

Bovine mastitis is one of the most detrimental diseases affecting the dairy industry worldwide, leading to substantial economic losses due to decreased milk yield and quality, altered milk composition, increased treatment costs, impaired fertility, and premature culling [1, 2]. Subclinical mastitis, in particular, represents a major economic burden owing to its high prevalence, prolonged course, and negative impact on milk production and quality, despite the absence of visible clinical signs [3]. Although mastitis may result from various factors, bacterial infection remains the predominant cause [4]. Mastitis-causing bacteria are commonly classified as contagious or environmental pathogens based on their epidemiology and transmission routes [5]. Environmental pathogens are particularly challenging to control because they persist in farm surroundings and can access the mammary glands during routine activities such as milking and resting [6].

*E. coli* is the most common environmental pathogen that can induce clinical and subclinical mastitis in dairy cows, causing significant losses to the dairy industry [6, 7]. Mastitis caused by *E. coli* is of particular concern because of its high prevalence, increasing antimicrobial resistance, and frequent involvement in intramammary infections during the dry period [8]. Antimicrobial therapy is widely used for mastitis prevention and treatment; however, inappropriate and excessive antimicrobial use has contributed to the emergence and spread of antimicrobial-resistant (AMR) bacteria, posing a serious challenge to animal health and food safety [9, 10]. The World Health Organization has identified *E. coli* as an important indicator organism in the development and dissemination of antimicrobial resistance, given its ability to acquire and transfer resistance genes [11].

In addition to antimicrobial resistance, biofilm formation has been recognized as a virulence-associated trait that may contribute to the persistence of mastitis-causing pathogens and reduced treatment efficacy [12–14]. *E. coli*, a common dairy-associated pathogen, can readily persist within biofilms, particularly under conditions that mimic mastitis, where low glucose availability and fluctuating pH levels favor biofilm development [15]. In addition, biofilms formed on contaminated milking equipment can act as reservoirs for *E. coli*, facilitating its dissemination into bulk tank milk, thereby compromising milk quality and safety [16]. Biofilm-embedded bacteria are more difficult to eliminate, potentially favoring recurrent or chronic intramammary infections and complicating mastitis control at the farm level [17–18]. Nevertheless, the relationships between biofilm formation, antimicrobial resistance, bacterial phylogeny, and farm management practices remain complex and incompletely understood, particularly under field conditions.

Despite growing international interest, integrated studies combining biofilm phenotypes, genotypic markers (including virulence-associated genes), phylogenetic backgrounds, phenotypic antimicrobial resistance, and farm-level management factors are scarce in Egypt and in dairy systems with similar production and hygiene characteristics. Addressing this gap is essential for developing effective, context-specific mastitis control and antimicrobial stewardship strategies. Therefore, the present study aimed to isolate *E. coli* from milk samples of cows with subclinical mastitis and characterize the isolates with respect to phylogenetic grouping, selected virulence genes, antimicrobial resistance profiles, and biofilm-forming capacity, while evaluating their associations within different dairy farm management settings.

## Materials and methods

### Study area

Between January 2023 and November 2024, a field study was conducted on five private dairy farms located in different Egyptian governorates (Cairo-Alexandria, Gharbia, Fayum, Ismailia, and Giza). Farms were purposively selected to represent different geographic locations and herd size categories (small, medium, and large-scale) based on the classification described by Megersa et al. [19], as well as accessibility, availability of farm records, and owner consent. Dairy farms were classified as small-scale (two farms), medium-scale, and large-scale (three farms) based on herd size. At each farm, lactating cows were selected using a simple random sampling method; however, in Gharbia, all lactating cows were sampled because of the relatively small herd size, which allowed complete coverage and ensured sufficient detection of subclinical mastitis cases. In larger herds, random sampling was applied for logistical feasibility while maintaining the representativeness. Depending on the herd size, 15–70 cows were sampled per farm. A structured questionnaire was used to collect information on farm hygiene and management (Supplementary Table 1).

### Sampling

A total of 254 composite quarter milk samples were collected aseptically from cows with subclinical mastitis. Subclinical mastitis was identified based on the interpretation of the california mastitis test (CMT), in the absence of visible clinical signs of mastitis [20]. Approximately 10 mL of milk was aseptically collected from cow teats (composite milk from all functional teats) following the standard milk sample testing protocols established by the National Mastitis Council [21]. Before milk sample collection, we assessed the udder’s cleanliness, state, and appearance for gross contamination and aberrations. The udder was then thoroughly cleaned and wiped with a clean, dry towel. Each teat was disinfected with 70% alcohol, the first streams of foremilk were discarded, and milk was aseptically collected into sterile vials. All milk samples were stored at 4 °C until bacteriological isolation.

### Bacteriological isolation and identification

The milk samples were incubated for 18–24 h at 37 °C then a loopful of incubated milk was cultured on Eosin Methylene Blue (EMB) agar medium (Oxoid, UK). All plates were incubated at 37 °C for 24–48 h and examined for bacterial growth. *E. coli* colonies from each plate were subjected to biochemical identification according to Quinn et al. [22].

### Molecular identification of the isolated *E. coli* strains using PCR

Bacterial DNA extraction was performed using the boiling method according to Wani et al. [23]. Briefly, single colonies were inoculated into BHI broth at 37 °C overnight. From which, 1.5 ml inoculated broth was centrifuged for 10 min at 1200×g. The pellet containing the bacteria was resuspended in 150 µl of sterile distilled water. The bacteria were then subjected to lysis via boiling in a water bath for 10 min. Afterwards, the lysate was subjected to a centrifugation step again to get rid of cellular debris. The supernatant was used as a DNA template for PCR and stored at -20 °C. The highly conserved 16S ribosomal RNA (*16SrRNA*) gene was selected for the molecular identification of *E. coli* isolates using the oligonucleotide primer sequences (5`–3`) F: CCCCCTGGACGAAGACTGAC, R: ACCGCTGGCAACAAAGGATA according to Wang et al. [24]. DNA amplification was accomplished in a total 25 µL mixture volume and were subjected to initial denaturation for 5 min at 94 °C followed by 35 cycles of denaturation (94 °C, 30 s), annealing (62 °C, 30 s), and elongation (72 °C, 1 min). These cycles were followed by a single extention step at 72 °C for 5 min. *E. coli* (ATCC8739) reference strain and nuclease free water were utilized as positive control for the* 16SrRNA* gene of *E. coli* and negative control, respectively.

The amplified products at 401 bp were separated by electrophoresis through 1.5% agarose (wt/vol), stained with 0.5 µg/mL ethidium bromide, visualized under UV illumination, imaged with a GelDoc 1000 fluorescent imaging system (Bio-Rad), and analyzed by Gel-pro analyzer^®^ version 4 (Media Cybernetics, Silver Spring, MD, USA).

### Detection of virulence and biofilm encoding genes

Virulence factors and biofilm encoding genes (*eae*,* stx*_*1*_, *stx*_*2*_, *st*,* fimH*, and *luxS*) were investigated in *E. coli* strains using different PCR assays (Table 1). *E. coli* strains were classified into pathotypes based on virulence genes as follows: ETEC (*st*), EPEC (*eae*), STEC (*stx*_*1*_ and/or *stx*_*2*_), and AE-STEC (*eae*, *stx*_*1*_ and/or *stx*_*2*_). Isolates harboring enterotoxin genes (*st*) together with *stx* genes were classified as ETEC–STEC hybrid strains (ETST) according to Rodwell et al. [25].

### Phylogrouping of *E. coli* strains

Molecular phylogrouping of different *E. coli* strains was performed using the Clermont method, based on the presence or absence of three specific genes (*chuA*, *yjaA*, and *TspE4.C2*) (Table 1). The phylogenetic grouping method can assign *E. coli* strains into four phylogroups, including A (*chuA*-/*TspE4.C2*-), B1 (*chuA*-/*TspE4.C2* +), B2 (*chuA* + /yjaA +), and D (*chuA* + /yjaA-) according to Clermont et al. [26].

**Table 1:** Primers used for virulence genes, biofilm-associated genes, and phylogroup determination of *Escherichia coli*

Target gene		Oligonucleotide sequences (5′–3′)	Product size (bp)	PCR condition	References
Pathotype (Virulence factors encoding genes)					
EPEC	*eae*	F: GTGGCGAATACTGGCGAGACT R: CCCCATTCTTTTTCACCGTCG	891	1 cycle (94 °C, 3 min) 30 cycles (94 °C, 30 s/55°C, 30 s/72°C, 60 s) 1 cycle (72 °C, 10 min)	Hornitzky et al. [27]
STEC	*stx*_*1*_	F: AAATCGCCATTCGTTGACTACTTCT R: CAGTCGTCACTCACTGGTTTCATCA	370		
	*stx*_*2*_	F: TGCCATTCTGGCAACTCGCGATGCA R: GGATCTTCTCCCCACTCTGACACC	283		
ETEC	*st*	F: TGT CTT TTT CAC CTT TCG CTC R: CGG TAC AAG CAG GATTAC AACAC	171		Müller et al. [28]
Biofilm encoding genes					
* fimH*		F: TGC AGA ACG GAT AAG CCG TGG R: GCA GTC ACC TGC CCT CCG GTA	508	1 cycle (95 °C, 5 min) 25 cycles (94 °C, 30 s/63°C, 30 s/68°C, 3 min) 1 cycle (72 °C, 10 min)	Johnson and Stell [29],
* luxS*		F: ATG CCG TTG TTA GAT AGC TTC A R: GAT GTG CAG TTC CTG CAA CTT C	513	1 cycle (95 °C, 5 min) 35 cycles (95 °C, 30 s/55°C, 30 s/72°C, 60 s) 1 cycle (72 °C, 5 min)	Wang et al. [30]
Phylogroup encoding genes					
* chuA*		F: GAC GAA CCA ACG GTCAGG AT R: TGC CGC CAG TAC CAAAGA CA	279	1 cycle (94 °C, 5 min) 30 cycles (94 °C, 30 s/55°C, 30 s/72°C, 30s) 1 cycle (72 °C, 7 min)	Clermont et al. [26]
* yjaA*		F: TGA AGT GTC AGG AGA CGC TG R: ATG GAG AAT GCG TTC CTC AAC	211		
* TspE4.C2*		F: GAG TAA TGT CGG GGCATT CA R: CGC GCC AAC AAA GTATTA CG	152		

*F* forward primer, *R* reverse primer, *bp* base pairs, *EPEC* enteropathogenic *Escherichia coli*, *ETEC* enterotoxigenic *Escherichia coli*, *STEC* Shiga toxin-producing *Escherichia coli*

Target genes: *eae* (intimin), *stx1 and stx2* (Shiga toxins 1 and 2), *st* (heat-stable enterotoxin), *fimH* (type 1 fimbrial adhesin), *luxS (*autoinducer-2 synthase), *chuA*,* yjaA*,* and TspE4.C2* (phylogrouping per Clermont method)

### Antibiotic susceptibility test

Isolate susceptibility to antibiotics was determined using the disc-diffusion method based on the measurement of the inhibition zones on Mueller-Hinton agar (Hi Media, India) according to the Kirby-Bauer method [31] and interpreted based on CLSI [32]. An antibiotic panel of both β-lactams and non-β-lactams (HiMedia, India) was used, including ampicillin (AMP, 10), amoxicillin/clavulanic acid (AMC, 20/10), cefuroxime (CXM, 30), ceftriaxone (CRO, 30), cefepime (CPM, 30), tetracycline (TE, 30), sulpha/trimethoprim (Co-Trimoxazole, COT, 25), ciprofloxacin (CIP, 5), nalidixic acid (NA, 30), and gentamicin (CN, 10). Quality control for antimicrobial susceptibility testing was performed using the standard reference strain (*Escherichia coli* ATCC8739) in accordance with standard guidelines. Multidrug-resistant (MDR) strains were determined based on resistance to at least one antibiotic in three or more antimicrobial classes [33].

### Biofilm formation

#### Preparation of bacterial Inoculum

The confirmed *E. coli* strains were cultured overnight in Tryptic soya broth (TSB) at 37 °C. The overnight cultures were diluted 1:100 in fresh TSB supplemented with 1% glucose to enhance the biofilm formation [34].

#### Microtiter plate assay (MTP)

Biofilm formation was assessed using a 96-well polystyrene microtiter plate, as described by Christensen et al. [35] in triplicates, with some modifications. Briefly, 200 µL of the diluted bacterial suspension was added to each well of a sterile flat-bottomed 96-well plate in triplicate. Wells containing 200 µL broth only were assigned as a negative control, while positive control wells contain 200 µL of reference *E. coli* strain (ATCC8739). The plates were incubated at 37 °C for 24 h under static conditions. After incubation, the wells were gently washed three times with phosphate-buffered saline (PBS, pH 7.2) to remove planktonic and unattached cells, and then dried at 60 °C for 1 h in an inverted position. The adherent biofilm was fixed with 200 µL of 99% methanol for 20 min, air-dried, and stained with 0.1% crystal violet for 30 min. Excess stain was removed by washing twice with sterilized distilled water, and the plates were air-dried overnight. The bound dye was resolubilized with 200 µL of 99% ethanol, and the absorbance was measured at 570 nm using a microtiter plate reader.

Biofilm-forming ability was categorized based on the optical density (OD) readings into non-biofilm producer (OD ≤ ODc), weak biofilm producer (ODc < OD ≤ 2ODc), moderate biofilm producer (2ODc < OD ≤ 4ODc), and strong biofilm producer (OD > 4ODc), and the biofilm OD cut-off value (ODc) = average negative control OD + (3 standard deviations (SD) of negative control) according to Stepanović et al. [36].

### Statistical analysis

Statistical analyses were performed using IBM SPSS Statistics v23 (SPSS Inc., Chicago, IL, USA) and R software. Data were summarized as frequencies and percentages for the categorical variables. Biofilm biomass was quantified using a crystal violet microtiter plate assay and expressed as OD. Assays were performed in triplicate, and the mean OD for each isolate was used for analysis. The normality of the OD570 data was assessed using the Shapiro–Wilk test. Comparisons of OD values between groups (e.g., biofilm strength categories and antimicrobial susceptibility profiles: susceptible/intermediate/resistant, MDR vs. non-MDR, and phylogroups were conducted using the Kruskal–Wallis test, followed by Dunn’s post hoc test with multiplicity-adjusted corrected p-values for multiple pairwise comparisons. Associations between categorical variables (e.g., biofilm strength categories versus phylogroups, antimicrobial resistance categories, and virulence gene presence/absence) were assessed using Pearson’s chi-square test. Hierarchical clustering of antibiotic resistance variables and isolates was performed using Ward’s linkage on Euclidean distances and visualized as a heat map. All tests were two-tailed, and statistical significance was set at *P* < 0.05.

To clarify the statistical hierarchy, our analyses were categorized into exploratory and confirmatory approaches. Hierarchical clustering (visualized via heat maps) was utilized as an exploratory method to identify natural groupings and visualize phenotypic patterns in antibiotic resistance across biofilm strengths and phylogroups. In contrast, Kruskal–Wallis tests (followed by Dunn’s post hoc tests) and Pearson’s chi-square tests were employed as confirmatory analyses to formally test for statistically significant differences and associations among these variables.

## Results

### Occurrence of *E. coli* in milk samples from cows with subclinical mastitis

A total of 254 composite milk samples were collected from cows with subclinical mastitis across five dairy farms located in the Cairo-Alexandria, Gharbia, Fayum, Ismailia, and Giza Governorates. *Escherichia coli* was isolated from 68 samples, with an overall prevalence of 26.7%. The isolation rates varied considerably across farms as shown in (Table 2).

**Table 2:** Prevalence of *E. coli* Isolated from Milk Samples of Cows with Subclinical Mastitis in Different Governorates

Governorate	Examined Samples	Positive Samples	Prevalence (%)
Cairo-Alexandria	43	13	30.2
Gharbia	15	3	20.0
Fayum	59	10	16.9
Ismailia	67	16	23.8
Giza	70	26	37.1
Total	254	68	26.7

### Molecular characteristics and pathotype distribution

Among the 68 *E. coli* isolates characterized (Table 3), both phenotypic and genotypic features were systematically analyzed, revealing notable patterns across various parameters. At the molecular level, all isolates harbored the *luxS* gene, whereas the *fimH* gene was detected in 80.9% of cases. In terms of pathotype distribution, the majority were non-pathogenic (82.4%), whereas smaller fractions carried ETEC (7.4%), EPEC (4.4%), STEC (2.9%), and ETST (2.9%) markers.

**Table 3:** Distribution of *E. coli* virulence genes and pathotypes (*n* = 68 isolates)

Variable	Category	Count	Percentage (%)
*FimH*	Negative (N)	13	19.1
	Positive (P)	55	80.9
	Total	68	100
*luxS*	Positive (P)	68	100
	Total	68	100
Pathotype	ETEC	5	7.4
	EPEC	3	4.4
	STEC	2	2.9
	ETST	2	2.9
	Non-pathogenic	56	82.4
	Total	68	100
*st*	Negative (N)	61	89.7
	Positive (P)	7	10.3
	Total	68	100
*stx*_*1*_	Negative (N)	68	100
	Total	68	100
*stx*_*2*_	Negative (N)	64	94.1
	Positive (P)	4	5.9
	Total	68	100
*eae*	Negative (N)	65	95.6
	Positive (P)	3	4.4
	Total	68	100

Values are n (%) calculated out of all isolates (*n* = 68)

Gene status: *P* positive, *N* negative

Pathotype assignment: ETEC (*st*-positive), EPEC (*eae*-positive), STEC (*stx1*- and/or *stx2*-positive), ETST (ETEC–STEC hybrid; *st* with *stx1* and/or *stx2*). “Non-pathogenic” denotes isolates negative for the tested diarrheagenic *E. coli* (DEC) virulence markers

### Molecular phylogrouping of *E. coli* strains

Phylogenetic grouping classified the *E. coli* strains into five groups: A, B1, B2, D, and untypeable, with group B1 predominating at approximately 51.9%, followed by group A (35.3%), with smaller proportions assigned to groups B2 (13.2%), D (1.5%), and untypeable strains (4.4%).

### Antibiotic resistance profiles and multidrug resistance

The antibiotic susceptibility patterns of *E. coli* strains were examined to determine the resistance prevalence and multidrug resistance (MDR) status (Table 4). Antimicrobial susceptibility testing revealed diverse resistance patterns against commonly used veterinary antibiotics.

Resistance rates were highest for amoxicillin/clavulanic acid (60.3%) and cefuroxime (58.8%), whereas all isolates were sensitive to cefepime (100%). Gentamicin resistance was low (3.7%), with some isolates showing intermediate resistance. Multidrug resistance was observed in 52.9% of the isolates. Most MDR isolates exhibited resistance to three antibiotic classes, mainly amoxicillin/clavulanic acid, ampicillin, tetracycline, and sulpha/trimethoprim.

**Table 4:** Antimicrobial Resistance Patterns of the isolated *E. coli* Strains (*n* = 68)

Antibiotic	Concentration (µg)	Resistant *n* (%)	Intermediate *n* (%)	Sensitive *n* (%)
Amoxicillin/Clavulanic Acid	20/10	41 (60.3)	-	27 (39.7)
Ampicillin	10	38 (55.9)	3 (4.4)	27 (39.7)
Cefuroxime	30	40 (58.8)	-	28 (41.2)
Ceftriaxone	30	13 (19.1)	1 (1.5)	54 (79.4)
Cefepime	30	0	-	68 (100.0)
Tetracycline	30	33 (48.5)	-	35 (51.5)
sulpha/trimethoprim	25	13 (19.1)	5 (7.4)	50 (73.5)
Enrofloxacin	10	3 (4.4)	-	65 (95.6)
Nalidixic Acid	30	1 (1.5)	3 (4.4)	64 (94.1)
Gentamicin	10	12 (17.6)	10 (14.7)	46 (67.6)

Values are n (%). "—" indicates zero isolates

Susceptibility interpretation followed CLSI M100 criteria. MDR was defined as resistance to at least one agent in three or more antimicrobial classes

### Biofilm formation capacity of *E. coli* strains 

The biofilm-forming ability of *E. coli* strains was quantified using the microtiter plate assay, with OD measured at 570 nm. Among the 68 isolates tested, moderate biofilm producers constituted the largest group (44.1%), followed by strong (38.2%) and weak producers (17.6%). The distribution of biofilm-forming capacity varied markedly among the farms (Table 5). Fayum and Ismailia showed the highest proportions of strong biofilm producers, with 100% and 75%, respectively, whereas Cairo-Alexandria and Giza predominantly harbored moderate biofilm producers.

**Table 5:** Distribution of biofilm formation capacity of *E. coli* strains by farm location

Farm location	Strong (*n*, %)	Moderate (*n*, %)	Weak (*n*, %)
Cairo-Alexandria	1 (7.7%)	8 (61.5%)	4 (30.8%)
Gharbia	1 (33.3%)	2 (66.7%)	0 (0%)
Fayum	10 (100%)	0 (0%)	0 (0%)
Ismailia	12 (75.0%)	4 (25.0%)	0 (0%)
Giza	2 (7.7%)	16 (61.5%)	8 (30.8%)
**Total**	26 (38.2%)	30 (44.1%)	12 (17.6%)

Values are n (% within farm)

Biofilm strength categories per Stepanović et al.: weak (ODc < OD ≤ 2ODc), moderate (2ODc < OD ≤ 4ODc), and strong (OD > 4ODc), where OD = optical density at 570 nm and ODc = mean OD of negative control + 3 SD

### Hierarchical clustering of antibiotic resistance and biofilm formation strength

**Fig. 1:**
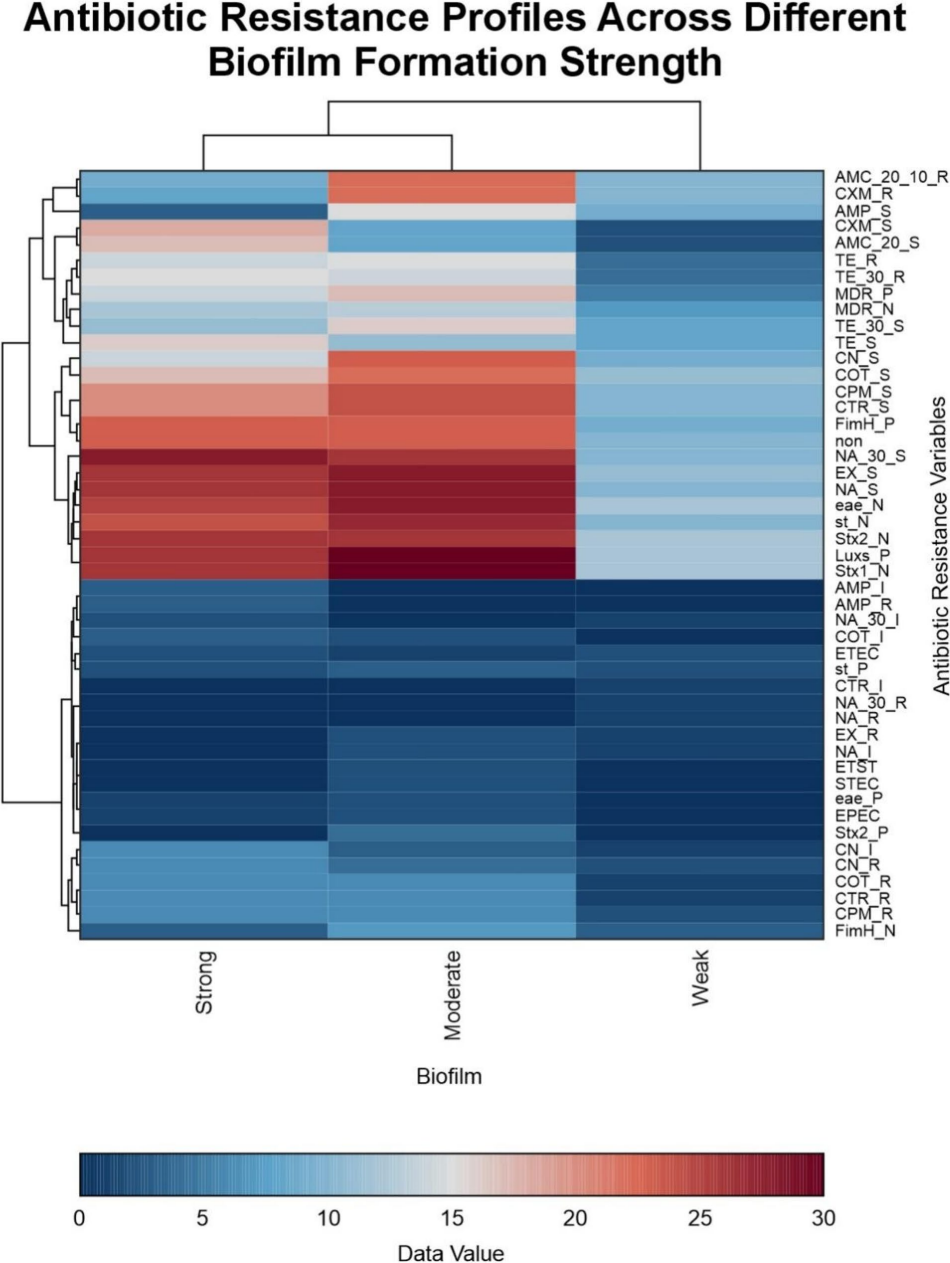
Clustered heat map of antibiotic resistance profiles across biofilm formation strengths. The heat map illustrates hierarchical clustering of antibiotic resistance variables and multidrug resistance (MDR) status (rows) across all 68 individual *E. coli* isolates (columns). The isolates are grouped along the x-axis by their respective biofilm categories: strong, moderate, and weak. Clustering of the antibiotic variables (dendrogram on the left) was performed using Ward’s linkage on Euclidean distances to show similarities in resistance patterns. Colors represent the specific phenotypic status of each isolate: blue shades indicate susceptibility (S) or a negative (N) MDR status, and red shades indicate resistance (R) or a positive (P) MDR status. Intermediate (I) susceptibility is represented by transitional shades according to the color scale bar. Abbreviations: AMC, amoxicillin/clavulanic acid; CXM, cefuroxime; AMP, ampicillin; TE, tetracycline; MDR, multidrug resistance; COT, co-trimoxazole; CN, gentamicin; EX, enrofloxacin; CTR, ceftriaxone; CPM, cefepime; NA, nalidixic acid. Scale Bar: Red = Resistant (R) / Positive (P) Blue = Susceptible (S) / Negative (N) White/Lighter shades = Intermediate (I)

To explore potential relationships between biofilm formation strength categories (weak, moderate, and strong biofilm producers) and antimicrobial resistance, hierarchical clustering analyses were performed (Fig. 1). The heat map revealed distinct clusters demonstrating that strong biofilm producers frequently exhibited elevated resistance to specific beta-lactams, such as amoxicillin/clavulanic acid and cefuroxime, compared with weak biofilm producers. However, statistical analyses confirmed that overall multidrug resistance (MDR) status did not significantly correlate with biofilm strength. This indicates that while increased biofilm capacity is associated with resistance to certain individual antibiotics, it is not a consistent predictor of a broader multidrug-resistant phenotype.

### Hierarchical clustering of antibiotic resistance and phylogenetic distribution

**Fig. 2:**
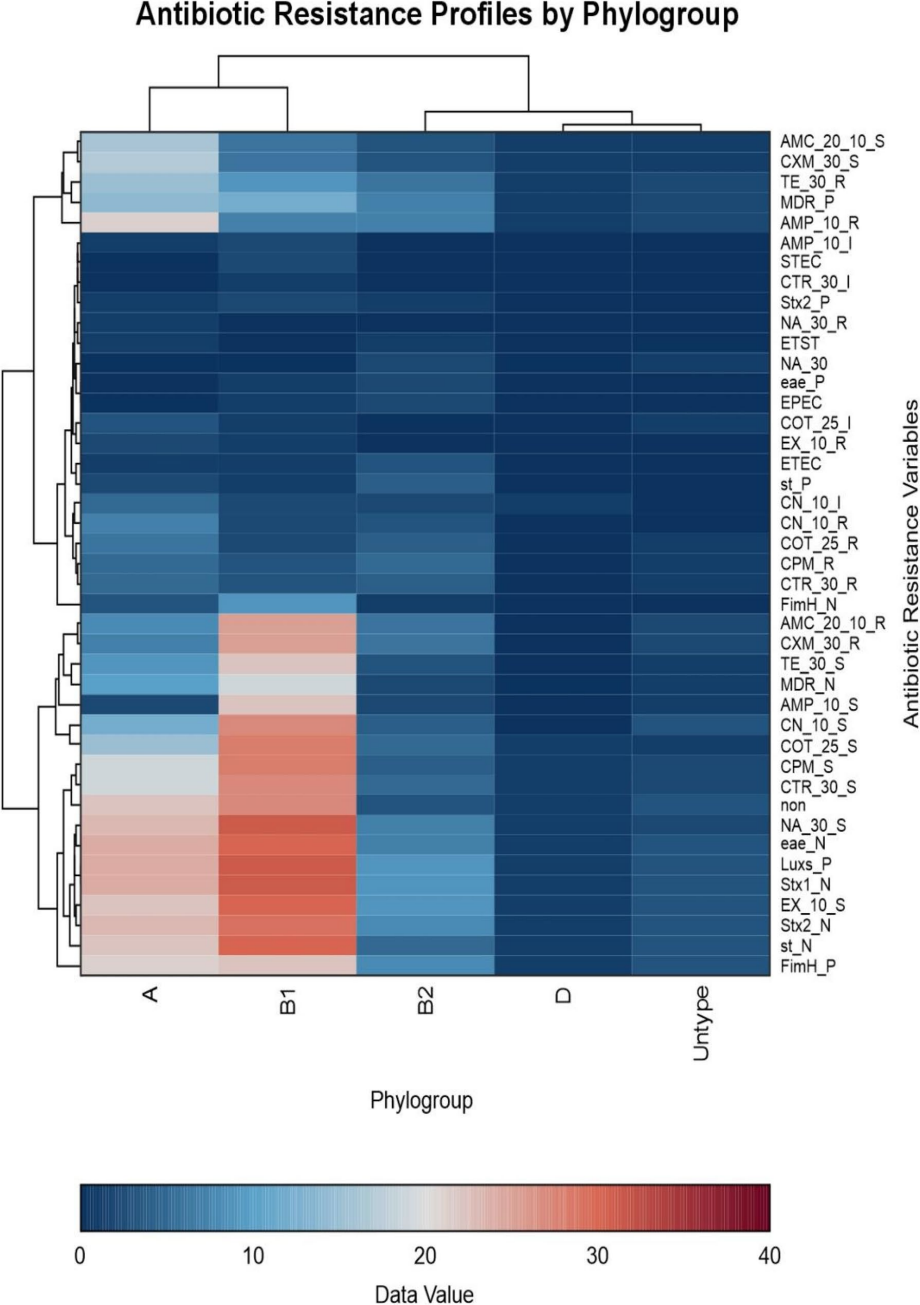
Clustered heat map of antibiotic resistance profiles across phylogroups. The heat map displays hierarchical clustering of antibiotic resistance variables (rows) across all 68 individual *E. coli* isolates (columns). The isolates are grouped along the x-axis by their assigned phylogroups: A, B1, B2, D, and untyped. Clustering of the antibiotic variables (dendrogram on the left) was performed using Ward’s linkage on Euclidean distances to show similarities in resistance patterns. Colors represent the specific phenotypic status of each isolate: blue shades indicate susceptibility (S) or a negative (N) status, and red shades indicate resistance (R) or a positive (P) status. Intermediate (I) susceptibility is represented by transitional shades. Abbreviations: R, resistant; S, susceptible; I, intermediate; P, positive; N, negative. Abbreviations: AMC, amoxicillin/clavulanic acid; CXM, cefuroxime; AMP, ampicillin; TE, tetracycline; MDR, multidrug resistance; COT, co-trimoxazole; CN, gentamicin; EX, enrofloxacin; CTR, ceftriaxone; CPM, cefepime; NA, nalidixic acid. Scale Bar: Red = Resistant (R) / Positive (P) Blue = Susceptible (S) / Negative (N) White/Lighter shades = Intermediate (I)

Distinct differences in antibiotic resistance profiles were observed among the different phylogroups. These patterns were supported by the hierarchical clustering of antibiotic resistance profiles across phylogroups (Fig. 2), which highlighted a prominent cluster of resistant phenotypes within B1 group, with group A displaying intermediate resistance. Groups B2, D, and Untyped clustered together, with predominantly susceptible profiles.

Although biofilm formation strength was not directly displayed in the heat map (Fig. 2), previous analyses indicated a stronger biofilm-forming capacity among isolates in groups A and B1 than in groups B2 and D.

### Biofilm formation optical density (OD) in relation to biofilm strength and antibiotic resistance profiles

**Fig. 3:**
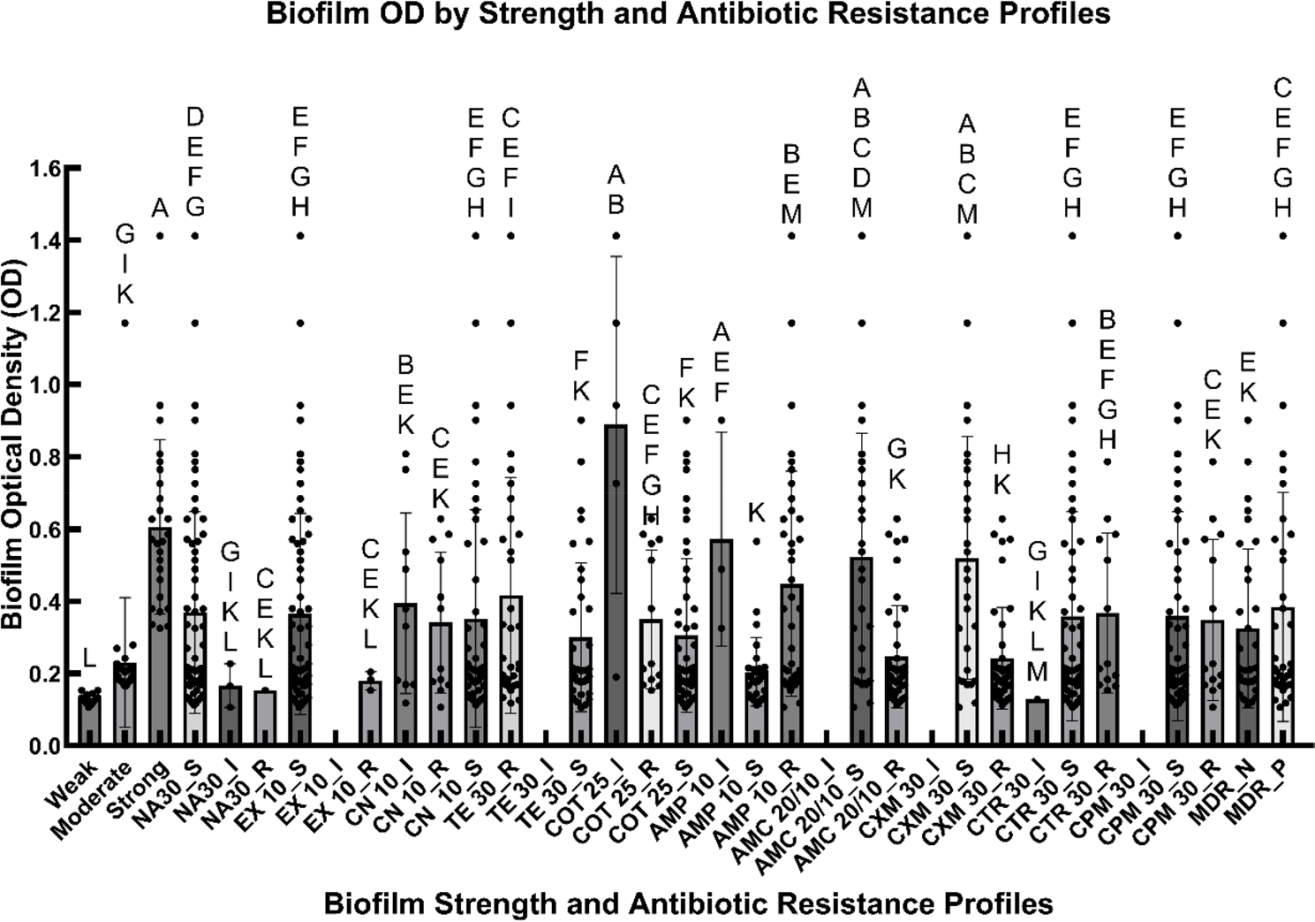
Optical density of biofilm formation according to biofilm-strength class and antibiotic resistance profile. Bars represent mean OD values, and error bars indicate standard deviation. Overlaid scatter dots represent individual isolates. Uppercase letters above bars indicate results of multiple-group comparisons; groups with different letters differ significantly at $\:p<0.05$, whereas groups sharing at least one letter do not differ significantly. Statistical significance was determined using Kruskal–Wallis test, followed by Dunn’s post hoc test

Biofilm formation, as assessed by OD, varied among the biofilm strength categories and across the antibiotic resistance profiles (Fig. 3). Strong biofilm producers exhibited significantly higher mean OD values than moderate and weak producers (*p* < 0.05), confirming the validity of the phenotypic classification derived from the microtiter plate assay results. When stratified by antimicrobial resistance profiles, isolates resistant to β-lactam antibiotics, including amoxicillin/clavulanic acid and cefuroxime, generally exhibited lower mean OD values than susceptible isolates, except for ampicillin. A similar tendency was observed across other antibiotic classes, whereby resistant isolates showed reduced biofilm biomass relative to susceptible and intermediate groups; however, these differences were not consistently statistically significant, as indicated by the grouping annotations in Fig. 3. Resistance to nalidixic acid was associated with slightly lower mean OD values, although the difference was marginal. No resistance to cefepime was detected among the examined isolates; therefore, no variation in biofilm formation could be evaluated for this antibiotic. Multidrug-resistant (MDR) isolates demonstrated a slightly higher mean biofilm OD than non-MDR isolates; however, this difference was not statistically significant (*p* > 0.05). Consistent with the chi-square and correlation analyses, these findings indicate that variations in biofilm formation may occur among isolates with different resistance profiles; however, our data do not establish a direct or statistically robust link between overall MDR status and enhanced biofilm production capacity in these *E. coli* isolates.

### Biofilm formation optical density (OD) variation across phylogroups and strains

**Fig. 4:**
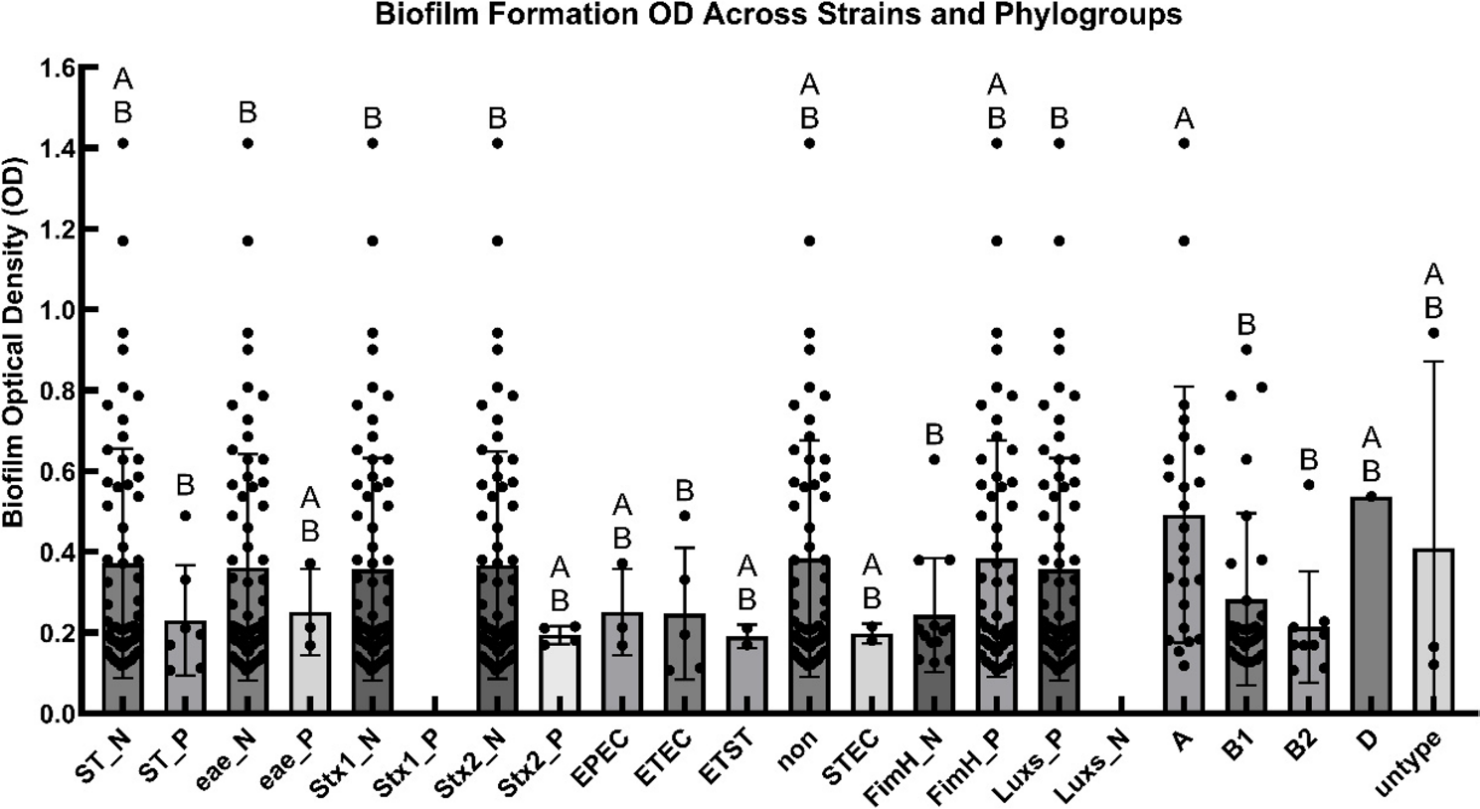
Optical density of biofilm formation according to virulence gene status, pathotype, and phylogroup. Bars represent mean OD values, and error bars indicate standard deviation. Overlaid dots represent individual isolates within each category. For gene-based categories, N indicates gene-negative isolates and P indicates gene-positive isolates. EPEC, ETEC, and ESTT represent pathotype groups; non and STEC indicate non-STEC and STEC isolates, respectively; *FimH* and *LuxS* indicate isolates classified by the presence or absence of these genes; and A, B1, B2, D, and untype represent phylogroup classifications. Different letters above bars indicate statistically significant differences at $\:p<0.05$; groups with different letters differ significantly, whereas groups sharing the same letter do not. Statistical analysis was performed using Kruskal–Wallis test, followed by Dunn’s post hoc test

Optical density measurements of biofilm formation revealed significant variability among *E. coli* phylogroups and individual strains within each phylogroup **(**Fig. 4**).** Phylogroup A isolates produced significantly higher biofilm biomass than other phylogroups, such as B1 and B2 (*p* < 0.05), consistent with their elevated antibiotic resistance profiles. This finding suggests a potential link between phylogenetic background and biofilm-forming capacity. Additionally, strain-level differences within phylogroups underscored considerable heterogeneity in biofilm production, indicating that biofilm formation is influenced not only by phylogroup classification but also by the characteristics of individual strains.

### Associations between biofilm formation and microbial, genetic, and environmental factors

The factors influencing biofilm strength among *E. coli* from subclinical mastitis using chi-square (Table 6) and correlation analyses (Table 7) were evaluated. Resistance to several beta-lactams was significantly associated with stronger biofilm formation (AMC 20/10: χ² = 11.952, *p* = 0.0025; AMP 10: χ² = 18.900, *p* = 0.0008; CXM 30: χ² = 14.032, *p* = 0.0009), whereas no significant associations were observed for gentamicin, sulpha/trimethoprim, cefepime, or ceftriaxone. Phylogroup was significantly related to biofilm strength (χ² = 16.471, *p* = 0.0361), with phylogroup A producing stronger biofilms, and farm origin showed a very strong effect (χ² = 42.857, *p* < 0.0001), implicating environmental/management factors. Extended chi-square analyses (Supplementary Tables 2–4) confirmed strong links between pathotypes and key virulence genes (*eae*,* st*, *stx*_*2*_; χ² = 68.000, *p* < 0.0001), and showed farm origin aligned with pathotype distribution and resistance to AMC, AMP, and CXM; phylogroups correlated with multiple resistance profiles and with farm origin but not with *FimH*, *luxS*, or MDR status.

Correlation analyses (Table 7) reinforced these relationships: biofilm classification correlated strongly with phenotypic OD cut-offs (contingency coefficient = 0.816) and moderately with phylogenetic background (Phi = 0.492; Cramer’s V = 0.348) and resistance to AMP (Phi = 0.527), AMC (Phi = 0.419), and CXM (Phi = 0.454); pathotype showed a moderate correlation (Phi = 0.349). Farm origin yielded the strongest correlations in supplementary analyses, whereas correlations between biofilm strength and other antibiotics (gentamicin, sulpha/trimethoprim, tetracycline, nalidixic acid), MDR status (Phi = 0.108), and several virulence markers (*eae*, *stx*_*2*_, *FimH*) were weak or absent. Collectively, these results indicate that enhanced biofilm formation among mastitis *E. coli* is chiefly associated with specific beta-lactam resistance, phylogenetic lineage, and farm/environmental factors, while MDR status and many virulence genes are poor predictors of biofilm capacity.

**Table 6:** Chi-square tests for the association between biofilm strength and microbial, genetic, and environmental classifications

Classification	Chi-square (χ²)	*p*-value	Significance
Farm	42.857	0.0001	Highly significant
Pathotype	8.277	0.4069	Not significant
*st*	0.721	0.6973	Not significant
*eae*	0.935	0.6265	Not significant
*Stx*_*1*_	N/A		N/A
*Stx*_*2*_	5.383	0.0678	Not significant
OD-ODC	136.000	0.3417	Not significant
*FimH*	1.579	0.4540	Not significant
*luxS*	N/A	N/A	N/A
Phylogroup	16.471	0.0361	Significant
NA 30	6.824	0.1455	Not significant
EX 10	1.999	0.3680	Not significant
CN 10	3.467	0.4103	Not significant
TE_30	2.025	0.3633	Not significant
COT 25	3.248	0.5172	Not significant
AMP 10	18.900	0.0008	Highly significant
AMC 20/10	11.952	0.0025	Highly significant
CXM 30	14.032	0.0009	Highly significant
CTR 30	5.667	0.2254	Not significant
CPM 30	0.218	0.8969	Not significant
MDR	0.788	0.6744	Not significant

Reported are Pearson’s chi-square statistics (χ²) and p-values. Significance threshold: *p* < 0.05

Significance categories: Highly significant (*p* ≤ 0.001), Significant (0.001 < *p* < 0.05), Not significant (*p* ≥ 0.05)

N/A (Not Applicable): Calculation not possible due to zero variance or zero counts in specific categories

Antibiotic codes correspond to those defined in Table 3. OD_ODC reflects internal consistency with phenotypic OD cut-offs

Disc contents (µg): AMP 10, ampicillin; AMC 20/10, amoxicillin/clavulanic acid; CXM 30, cefuroxime; CRO 30, ceftriaxone; CPM 30, cefepime; TE 30, tetracycline; COT 25, co-trimoxazole (sulfamethoxazole/trimethoprim); CIP 5, ciprofloxacin; EX 10, enrofloxacin; NA 30, nalidixic acid; CN 10, gentamicin

**Table 7:** Correlation of biofilm formation with key variables

Variable	Phi	Cramer’s V	Pearson’s contingency coefficient	Kendall’s tau-B	Gamma	Interpretation
Pathotype	0.349	0.247	0.329	0.039	0.196	Moderate
*st*	0.304	0.215	0.290	0.018	0.139	Weak
*eae*	0.117	0.117	0.116	-0.025	-0.509	Weak/Negative
*Stx*_*1*_	N/A	N/A	N/A	N/A	N/A	Not available
*Stx*_*2*_	0.281	0.281	0.271	-0.067	-1.000	Weak/Negative
OD_ODC	1.000	1.000	0.816	0.025	0.040	Very Strong
Biofilm	1.000	1.000	0.816	0.637	1.000	Perfect
*FimH*	0.152	0.152	0.151	0.019	0.096	Weak
*luxS*	N/A	N/A	N/A	N/A	N/A	Not available
Phylogroup	0.492	0.348	0.442	-0.068	-0.156	Moderate
NA__30	0.317	0.224	0.302	-0.015	-0.187	Weak
EX_10	0.171	0.171	0.169	0.009	0.156	Weak
CN_10	0.242	0.171	0.235	-0.051	-0.160	Weak
TE_30	0.173	0.173	0.170	0.017	0.052	Weak
COT_25	0.219	0.155	0.214	0.037	0.143	Weak
AMP_10	0.527	0.373	0.466	-0.021	-0.057	Moderate
AMC_20_10	0.419	0.419	0.387	0.050	0.150	Moderate
CXM_30	0.454	0.454	0.414	0.058	0.169	Moderate
CTR_30	0.289	0.204	0.277	-0.002	-0.008	Weak
CPM_30	0.057	0.057	0.056	0.004	0.017	Very Weak
MDR	0.108	0.108	0.107	-0.050	-0.157	Very Weak

## Discussion

*E. coli* is one of the most notable environmental pathogens responsible for bovine mastitis [37, 38]. In the present study, *E. coli* isolates from subclinical bovine mastitis cases on five Egyptian dairy farms were comprehensively characterized using phenotypic and genotypic approaches, including virulence gene profiling, phylogrouping, antimicrobial susceptibility testing, and biofilm formation assays. *E. coli* was isolated from 68 out of 254 composite milk samples, representing a prevalence of 26.7% among cows with subclinical mastitis. This prevalence aligns with previous reports indicating the frequent involvement of *E. coli* in mastitis cases, although it was lower than the 49.8% prevalence reported by Abed et al. [39] in Upper Egypt. Variations in prevalence may be attributed to differences in herd management practices, environmental hygiene, and geographical conditions. Similar studies have identified *E. coli* as the predominant pathogen in subclinical mastitis, highlighting its widespread distribution in dairy farm environments [40]. Collectively, these findings emphasize the ongoing challenge posed by *E. coli* in dairy herds and underscore the importance of implementing effective management and hygiene measures to reduce the incidence of subclinical mastitis and minimize milk contamination [41].

Regarding farm-level distribution, the highest *E. coli* isolation rate was recorded in Giza (37.1%), whereas the lowest was observed in Fayum (16.9%). The significant variation in isolation rates among farms likely reflects differences in management practices and environmental conditions that influence the survival and transmission of bacteria. Heterogeneity in *E. coli* prevalence has been widely reported and is often associated with factors such as antimicrobial usage patterns, inadequate manure removal, suboptimal hygiene practices, water source management, and overall herd health status [39, 42, 43]. Consistent with this, Verbeke et al. [44] reported a 1.5-fold higher risk of mastitis in herds with dirty udders than in herds with good udder cleanliness. Additionally, dairy housing design and bedding management play a critical role in mastitis epidemiology, as cows remain in prolonged contact with bedding materials for approximately 12–14 h per day, facilitating teat contamination [45]. Understanding these farm-specific risk factors is crucial for developing targeted preventive strategies. Therefore, enhanced surveillance, improved hygiene, and tailored intervention programs are warranted in regions exhibiting high *E. coli* isolation rates to effectively mitigate the risk of mastitis in dairy herds [7].

There is no specific profile of the virulence factors implicated solely in *E. coli* associated with bovine mastitis [46]. This study investigated the lower rate of virulence genes associated with diarrheogenic *E. coli* (DEC) in the isolated strains. The *eae*,* st*, and *stx*_*2*_ genes were detected in 4.4%, 10.3%, and 5.9% of the isolated strains , respectively, with the absence of *stx*_*1*_ gene. This finding agrees with previous reports that detected milk STEC strains mainly carrying the *stx*_*2*_ gene [47–49]. Stx2-carrying phages are more inducible under antibiotic exposure and oxidative stress than Stx1 phages, which increase toxin expression and horizontal gene transfer of *stx*_*2*_ in cattle environments and contribute to a higher prevalence of this gene in cattle populations [50]. Several studies have found that *E. coli* isolated from cows with mastitis is less genotypically diverse than environmental strains and mostly lacks known *E. coli* virulence genes [51, 52]. The limited distribution of virulence genes suggests that mastitis-associated *E. coli* may have undergone selective pressure based on the presence of specific virulence factors associated with the ability to survive in milk [53, 54]. Based on the virulence genes studied, only 17.6% (12/68) were classified into different pathotypes, including ETEC (7.4%), EPEC (4.4%), STEC (2.9%), and a hybrid ETEC–STEC (2.9%). Ahmadi et al. [55] isolated STEC strains from bovine subclinical mastitis at 22.5%, with a higher rate of *stx*_*2*_ gene. In contrast, a previous study found that EPEC was widely present in mastitis-causing *E. coli* at 81.5%, while ETEC was detected at 6.5%, with the absence of STEC strains [56]. The presence of STX2-positive strains in milk from cows with subclinical mastitis underscores the potential zoonotic risk through the dairy food chain, particularly in regions where raw or inadequately pasteurized milk or dairy products are consumed.

The phylogenetic analysis confirmed that the isolates segregated mainly into phylogenetic groups B1 (51.9%) and A (35.3%), whereas phylogenetic groups B2 and D were present at comparatively lower rates of 13.2% and 1.5%, respectively. Similarly, several reports have shown that B1 and A are the dominant phylogenetic lineages among *E. coli* associated with bovine mastitis, which is regarded as commensal and environmental strains [6, 51, 57]. However, strains associated with these phylogroups may cause disease under circumstances of host susceptibility, such as immunosuppression or physiological stress, which impair natural defense mechanisms [58]. Furthermore, horizontal gene transfer through mobile genetic elements such as plasmids and transposons could promote the acquisition of virulence genes, increasing the pathogenic potential of these phylogroups [59]. Several investigations identified the virulence genes associated with phylogroups B1 and A in the isolated *E. coli* strains. For instance, previous findings reported that phylogroup B1 was the most prominant phylogroup within shiga toxin-producing *E. coli* (STEC) and enterohemorrhagic *E. coli* (EHEC) [60, 61]. Furthermore, Lan et al. [62] revealed that B1 and A phylogroups were predominated within enteropathogenic *E. coli* (EPEC) strains isolated from bovine mastitis. Additionally a prior study determined the virulence genes of extraintestinal *E. coli* within phylogroup B1 from acute bovine mastitis [63].

Antibiotics are crucial for curing serious cases of bovine mastitis caused by *E. coli* [64]. Cephalosporins, aminoglycosides, and tetracyclines are widely used to treat bovine bacterial mastitis [65]. In the present study, the isolated *E. coli* strains exhibited higher resistance to amoxicillin/clavulanic acid, ampicillin, cefuroxime, and tetracycline at 60.3%, 55.9, 58.8%, and 48.5%, respectively. In contrast, higher sensitivity was detected for cefepime, enrofloxacin, ceftriaxone, and gentamicin at 100%, 95.6%, 79.4%, and 67.6%, respectively. A comparable study determined the highest sensitivity against gentamicin and nalidixic acid at 97.5% and 80%, respectively, while resistance was frequently observed for tetracycline (30%) and sulpha/trimethoprim (27.5%) [66]. Several studies have reported lower resistance to gentamicin and ceftiofur in *E. coli* isolates from bovine mastitis [67, 68]. Furthermore, Hinthong et al. [69] observed resistance to cefuroxime, ceftriaxone, and gentamicin in 23% of isolates derived from the milk of cows with subclinical mastitis.

Multidrug resistance was observed in 52.9% of isolates, particularly for amoxicillin/clavulanic acid, ampicillin, tetracycline, and sulpha/trimethoprim. In contrast, Zuo et al. [6] and Ahmadi et al. [55] reported MDR rates of 79.48% and 36.8%, respectively. The overuse of antimicrobials in dairy farms and the presence of resistance genes on mobile genetic elements that can spread easily within commensal and pathogenic *E. coli* strains lead to the emergence of multidrug resistance in bovine mastitis [70, 71]. The presence of multidrug-resistant *E. coli* strains raises public health concerns because these bacteria can be transmitted to humans through contaminated dairy products or direct contact with animals [72].

Biofilm formation is an important pathogenic factor that enables bacteria to survive in the mammary glands and persist in the dairy herds [18]. It helps bacteria evade the host’s innate immune system, increases their resistance to antimicrobial agents, and hinders the treatment of recurrent infections [73]. In the present study, most of the isolated *E. coli* strains exhibited moderate and strong biofilm formation at 44.1% and 38.2%, respectively. This may be attributed to the prolonged survival of *E. coli* associated with subclinical mastitis in the udder, leading to persistent infections with strong biofilm formation [6]. There were variations in biofilm production among isolates from different farms, with higher rates of strong biofilm producers in Fayum and Ismailia at 100% and 75%, respectively. The hygienic conditions and management practices of dairy farms play an important role in determining the prevalence of strong biofilm producers on these farms. This suggests that environmental factors, such as management practices, sanitation methods, and water quality influence biofilm formation. Insufficient cleaning can result in the formation of biofilms on surfaces, as biofilms act as barriers to disinfectants [74]. By implementing effective management strategies, it is possible to mitigate biofilm formation, which is essential for ensuring product safety and quality. However, some studies suggest that biofilm formation is also possible in well-maintained environments, suggesting that factors other than hygiene, such as the genetic predisposition of the strains, may also contribute to biofilm development [75].

Regarding biofilm virulence genes, all the isolated *E. coli* strains harbored the *luxS* gene, whereas the *fimH* gene was detected in 80.9% of the cases. The *fimH* gene is an adhesion-encoding gene that encodes fimbrial adhesion (type 1 pili) and promotes bacterial colonization, invasion, and biofilm formation [76]. *luxS* is essential for regulating bacterial behavior and influencing *E. coli* biofilm development [77]. According to Gajdács et al. [78], biofilm formation was noted without significant virulence gene expression, and there was no correlation between biofilm genes and the virulence or resistance profiles.

In an investigation of the biofilm formation of *E. coli* strains isolated from mastitic milk, significant heterogeneity was observed among the strains of different phylogroups. Strains belonging to phylogroup A exhibited more biofilm production than those belonging to phylogroups B1 and B2. Based on this observation, there may be a possible association between the capacity for biofilm development and phylogenetic lineage. The observed variability in biofilm synthesis within individual phylogroups indicates that phylogenetic classifications and strain-specific traits may affect the phenotypic characteristics of biofilms. According to Nalband et al. [79], strains of phylogroup A from mastitic sources tend to produce greater quantities of biofilm biomass, a trait that our data links specifically to elevated beta-lactam resistance. Thus, genetic and phenotypic factors appear to be involved in biofilm regulation.

The heat map revealed distinct clusters demonstrating that strong biofilm producers frequently exhibited elevated resistance to specific beta-lactams, such as amoxicillin/clavulanic acid and cefuroxime, compared with weak biofilm producers. However, statistical analyses confirmed that overall multidrug resistance (MDR) status did not significantly correlate with biofilm strength. This indicates that while increased biofilm capacity is associated with resistance to certain individual antibiotics, it is not a consistent predictor of a broader multidrug-resistant phenotype in our strain collection. Therefore, genetic and environmental factors, rather than generalized multidrug resistance, appear to be the primary drivers of biofilm regulation in these isolates. A prior study reported that all *E coli* strains isolated from bovine mastitis were capable of forming biofilms, although at varying strengths [80]. The higher biofilm-forming ability of *E. coli* results in intrinsic resistance to several antimicrobials, contributing to the emergence of recurrent mastitis [13]. The distinctive characteristics of biofilms, including the impermeability, presence of persister cells, and the increased expression of efflux pumps assume a substantial role in the development of antimicrobial resistance [81]. Biofilms are constituted of structured bacterial communities incorporated within an extracellular polymeric substance (EPS) matrix which prohibits antibiotic penetration and slows molecular diffusion, reducing the penetration of antimicrobial drugs to the bacteria embedded in deeper layers. Furthermore, biofilms harbor persister cells that can survive antimicrobial exposure and subsequently regenerate the biofilm once antibiotic concentration is eliminated, making them an essential determinant of chronic and recurrent infections [82, 83]. Efflux pumps are crucial contributors to antimicrobial resistance in microbial pathogens associated with mastitis [84]. Biofilms frequently induce elevated expression of efflux pumps, which is a membrane protein complex that actively expel antimicrobial agents from the cell, lowering intracellular accumulation and leading to multidrug resistance [85].

While this study provides an integrated phenotypic and genotypic characterization of biofilm-forming *E. coli* associated with subclinical bovine mastitis across five Egyptian dairy farms, several limitations should be acknowledged. First, antimicrobial resistance was assessed phenotypically to provide an initial overview of resistance patterns among the isolated *E. coli* strains, while molecular characterization of AMR genes was not performed.

Consequently, the genetic mechanisms underlying observed resistance patterns, including the contribution of mobile genetic elements, could not be fully elucidated. Second, although key virulence-associated genes were investigated, the virulence genes panel was limited and may not capture the full spectrum of pathogenic determinants involved in mastitis pathogenesis. Third, the cross-sectional study design restricts interpretation to associations only and does not allow causal inferences regarding the relationships among biofilm formation, antimicrobial resistance, and farm management factors. Finally, the study focused exclusively on milk samples, and environmental sampling (e.g., bedding, water sources, milking equipment, and bulk tank milk) was not included, limiting insight into environmental reservoirs and transmission pathways of *E. coli* within dairy farm settings.

Future studies incorporating longitudinal designs, expanded virulence and resistance gene analyses, whole-genome sequencing, and comprehensive environmental sampling would strengthen understanding of *E. coli* ecology, persistence, and control in dairy herds.

## Conclusion

*E. coli* has a considerable impact on bovine subclinical mastitis in Egypt. The detection of diarrheagenic *E. coli* virulence genes occurred at low rates, without a significant association with biofilm formation and/or antimicrobial resistance. Moreover, the predominant phylogroups A and B1 and their significant correlation with increased biofilm formation and multidrug resistance illustrate the potential of commensal and environmental *E. coli* strains to produce persistent udder infection and cause antibiotic therapeutic failure. Multidrug-resistant strains exhibited resistance mainly to amoxicillin/clavulanic acid, ampicillin, tetracycline, and sulpha/trimethoprim, consistent with the selective pressure imposed by commonly used antimicrobials. However, our statistical analyses demonstrated that overall MDR status was not a significant predictor of biofilm production. Instead, enhanced biofilm formation was significantly associated with specific beta-lactam resistance, phylogenetic background (groups A and B1), and farm-level environmental factors. These observations underscore the importance of improved herd management practices, rigorous farm hygiene, and prudent antimicrobial use to reduce udder contamination with environmental *E. coli* and limit the emergence of biofilm-forming and antimicrobial-resistant strains in dairy herds.

## Supplementary Information

Supplementary Material 1.

## Data Availability

All data generated or analysed during this study are included in this published article and its supplementary information files.
