# Supplementary material for: Phenotypic and genotypic characterization of biofilm-forming Escherichia coli from subclinical bovine mastitis and association with antimicrobial resistance
Source: BMC Vet Res. 2026 Jun 3;22:338. doi: 10.1186/s12917-026-05598-2 (PMC13248448; doi:10.1186/s12917-026-05598-2)
Supplement: Supplementary file 1 — Supplementary Material 1. [file 12917_2026_5598_MOESM1_ESM.docx]

|  | Farm 1 | 2 | 3 | 4 | 5 |
| --- | --- | --- | --- | --- | --- |
| Study areas | Cairo-Alexandria | Gharbia | Fayum | Ismailia | Giza |
| Cow age | > 10 years | > 10 years | <10 y | <10 y | <10 y |
| The herd size | 45 animal | 15 animals | 70  animal | 90 animal | 150 animals |
| Milker’s hand washing | Sometimes | Mostly | Never | Mostly | Sometimes |
| Farm hygiene | Poor | Good | Good | Poor | moderate |
| Use of disinfectant | No | Yes | Yes | No | No |
| Udder washing | Yes | Mostly | No | Mostly | Sometimes |
| Washing hand between milking (yes/no) | No | No | Yes | No | No |
| Teat dip (yes/no) | No | Yes | Yes | No | No |
| Vaccination (yes/no) | No | Yes | Yes | No | Yes |

**Supplementary Table 1: The biosecurity level among dairy cattle farms in this study**

**Supplementary Table 2: Chi-square tests for association between Pathotype and various classifications**

| Classification Variable | Chi-square | P-value | Significance |
| --- | --- | --- | --- |
| AMC_20_10 | 4.285 | 0.3688 | Not significant |
| AMP_10 | 6.783 | 0.5603 | Not significant |
| Biofilm | 8.277 | 0.4069 | Not significant |
| CN_10 | 11.282 | 0.1862 | Not significant |
| COT_25 | 6.502 | 0.5912 | Not significant |
| CPM_30 | 6.341 | 0.1751 | Not significant |
| CTR_30 | 5.598 | 0.6922 | Not significant |
| CXM_30 | 4.361 | 0.3593 | Not significant |
| eae | 68.000 | 0.0001 | Highly significant |
| EX_10 | 0.673 | 0.9547 | Not significant |
| Farm | 33.030 | 0.0073 | Significant |
| FimH | 2.713 | 0.6070 | Not significant |
| Luxs | - | - | Not applicable |
| MDR | 2.593 | 0.6281 | Not significant |
| NA_30 | 3.500 | 0.8992 | Not significant |
| OD_ODC | 260.060 | 0.4873 | Not significant |
| Phylogroup | 26.107 | 0.0525 | Marginally NS |
| sample_no | 0 | - | Not applicable |
| st | 68.000 | 0.0001 | Highly significant |
| Stx1 | - | - | Not applicable |
| Stx2 | 68.000 | 0.0001 | Highly significant |
| TE_30 | 2.477 | 0.6488 | Not significant |

**Supplementary Table 3: Chi-square tests for association between Farms and various classifications**

| Classification Variable | Chi-square | P-value | Significance |
| --- | --- | --- | --- |
| AMC_20_10 | 33.845 | <0.0001 | Highly significant |
| AMP_10 | 51.646 | <0.0001 | Highly significant |
| Biofilm | 42.857 | <0.0001 | Highly significant |
| CN_10 | 14.924 | 0.0606 | Marginally NS |
| COT_25 | 11.715 | 0.1644 | Not significant |
| CPM_30 | 8.812 | 0.0660 | Marginally NS |
| CTR_30 | 7.793 | 0.4539 | Not significant |
| CXM_30 | 36.563 | <0.0001 | Highly significant |
| eae | 5.070 | 0.2802 | Not significant |
| EX_10 | 5.070 | 0.2802 | Not significant |
| FimH | 8.873 | 0.0643 | Marginally NS |
| Luxs | N/A | N/A | Not applicable |
| MDR | 7.112 | 0.1301 | Not significant |
| NA_30 | 14.834 | 0.0625 | Marginally NS |
| OD_ODC | 268.077 | 0.3521 | Not significant |
| Pathotype | 33.030 | 0.0073 | Significant |
| Phylogroup | 70.607 | <0.0001 | Highly significant |
| sample_no | N/A | N/A | Not applicable |
| st | 22.862 | 0.0001 | Highly significant |
| Stx1 | N/A | N/A | Not applicable |
| Stx2 | 4.087 | 0.3944 | Not significant |
| TE_30 | 6.515 | 0.1639 | Not significant |

**Supplementary Table 4: Chi-square tests for association between phylogroup and different variables.**

| Classification Variable | Chi-square | P-value | Significance |
| --- | --- | --- | --- |
| AMC_20_10 | 14.372 | 0.0062 | Significant |
| AMP_10 | 27.197 | 0.0007 | Highly significant |
| Biofilm | 16.471 | 0.0361 | Significant |
| CN_10 | 18.446 | 0.0181 | Significant |
| COT_25 | 14.372 | 0.0726 | Marginally NS |
| CPM_30 | 9.546 | 0.0488 | Significant |
| CTR_30 | 7.194 | 0.5158 | Not significant |
| CXM_30 | 16.543 | 0.0024 | Highly significant |
| eae | 8.165 | 0.0857 | Marginally NS |
| EX_10 | 1.579 | 0.8126 | Not significant |
| Farm | 70.607 | <0.0001 | Highly significant |
| FimH | 3.969 | 0.4102 | Not significant |
| Luxs | N/A | N/A | Not applicable |
| MDR | 6.144 | 0.1886 | Not significant |
| NA_30 | 17.084 | 0.0293 | Significant |
| OD_ODC | 264.612 | 0.4089 | Not significant |
| Pathotype | 26.107 | 0.0525 | Marginally NS |
| sample_no | N/A | N/A | Not applicable |
| st | 13.603 | 0.0087 | Significant |
| Stx1 | N/A | N/A | Not applicable |
| Stx2 | 0.840 | 0.9330 | Not significant |
| TE_30 | 9.234 | 0.0555 | Marginally NS |
